# Aged Skeletal Muscle Retains the Ability to Remodel Extracellular Matrix for Degradation of Collagen Deposition after Muscle Injury

**DOI:** 10.3390/ijms22042123

**Published:** 2021-02-20

**Authors:** Wan-Jing Chen, I-Hsuan Lin, Chien-Wei Lee, Yi-Fan Chen

**Affiliations:** 1 The Ph.D. Program for Translational Medicine, College of Medical Science and Technology, Taipei Medical University and Academia Sinica, Taipei 11529, Taiwan; d622105003@tmu.edu.tw; 2 TMU Research Center of Cancer Translational Medicine, Taipei Medical University, Taipei 11031, Taiwan; ycl6@tmu.edu.tw; 3 Institute for Tissue Engineering and Regenerative Medicine, The Chinese University of Hong Kong, Hong Kong, China; chienweilee@cuhk.edu.hk; 4 School of Biomedical Sciences, Faculty of Medicine, The Chinese University of Hong Kong, Hong Kong, China; 5 Graduate Institute of Translational Medicine, College of Medical Science and Technology, Taipei Medical University, Taipei 11031, Taiwan; 6 International Ph.D. Program for Translational Science, College of Medical Science and Technology, Taipei Medical University, Taipei 11031, Taiwan; 7 Master Program in Clinical Pharmacogenomics and Pharmacoproteomics, School of Pharmacy, Taipei Medical University, Taipei 11031, Taiwan

**Keywords:** aging, skeletal muscle, extracellular matrix (ECM), collagen, muscle injury

## Abstract

Aging causes a decline in skeletal muscle function, resulting in a progressive loss of muscle mass, quality, and strength. A weak regenerative capacity is one of the critical causes of dysfunctional skeletal muscle in elderly individuals. The extracellular matrix (ECM) maintains the tissue framework structure in skeletal muscle. As shown by previous reports and our data, the gene expression of ECM components decreases with age, but the accumulation of collagen substantially increases in skeletal muscle. We examined the structural changes in ECM in aged skeletal muscle and found restricted ECM degradation. In aged skeletal muscles, several genes that maintain ECM structure, such as transforming growth factor β (TGF-β), tissue inhibitors of metalloproteinases (TIMPs), matrix metalloproteinases (MMPs), and cathepsins, were downregulated. Muscle injury can induce muscle repair and regeneration in young and adult skeletal muscles. Surprisingly, muscle injury could not only efficiently induce regeneration in aged skeletal muscle, but it could also activate ECM remodeling and the clearance of ECM deposition. These results will help elucidate the mechanisms of muscle fibrosis with age and develop innovative antifibrotic therapies to decrease excessive collagen deposition in aged muscle.

## 1. Introduction

The ECM of skeletal muscle maintains tissue integrity and provides structural support [1,2]. Generally, there are three layers of ECM in skeletal muscle: the endomysium, perimysium, and epimysium [3,4]. The ECM comprises up to 10% of muscle weight and is composed of glycoproteins such as collagens, laminins, fibronectin and glycosaminoglycans (GAGs), short polysaccharide chains that bind to a protein core to form proteoglycans [5,6,7]. Interactions between collagen and proteoglycans maintain the ECM structure and organization.

Collagen is the main structural protein in the skeletal muscle ECM and is essential for the mechanical support of tissues. Fibrillar collagens (types I, II, III, V, XI, XXIV, and XXVII) provide three-dimensional structures for tissues [8]. More than 90% of fibrillar collagens are found in skeletal muscle and are mostly composed of collagens I, III, and IV [9]. Collagen I has been reported to be predominant in the perimysium, whereas collagen III is prevalent in the endomysium and the epimysium [2,10]. Networking collagens such as collagen IV are expressed mainly in the basement membrane (BM) [11,12], and collagens VI, XV, and XVIII are also present in the BM of skeletal muscle [13].

Skeletal muscle aging is characterized by the progressive loss of muscle mass and functions, which have been linked to the decreased regenerative potential of resident stem cells over time and increased vulnerability to diseases. Based on our current understanding, the aging of skeletal muscle can be caused by a combination of extrinsic and intrinsic factors [14]. Extrinsic factors include environmental changes such as modifications in the muscle stem cell (MuSC) niche, while intrinsic factors include biological changes in stem cells such as an impaired self-renewal capacity [15]. In mouse skeletal muscle, MuSCs are mitotically quiescent, expressing paired box 7 (Pax7) and CD34 [16]. Upon muscle injury, MuSCs activate, proliferate and fuse to form regenerated myofibers that facilitate repair via increased expression of certain myogenic regulation factor genes [17,18]. Proliferating satellite cells maintain the expression of Pax7 and induce the expression of myogenic differentiation 1 (MyoD1) and myogenic factor 5 (Myf5) [19,20]. A subset of these dividing satellite cells commits to differentiation, through the expression of Myogenin and myogenic factor 6 (Myf6), accompanied by the downregulation of Pax7, ultimately fusing to regenerated myofibers with centrally located nuclei [21,22]. On the other hand, another subset of activated satellite cells self-renews and re-instates quiescence to replenish the functional muscle stem cell pool for supporting future rounds of muscle repair. Alterations in the collagen network, an extrinsic factor, may contribute to the deterioration of muscle mechanical properties with aging. These structural and biochemical changes in the ECM of aged skeletal muscle may lead to increased stiffness and impairment of force generation by muscle fibers [4].

In this study, we analyzed ECM-related gene expression and collagen accumulation in young and aged skeletal muscles under normal and regenerative conditions. After muscle injury, ECM remodeling in both young and aged muscles occurs during muscle regeneration, allowing the clearance of deposited collagen. The possible mechanism for clearance was initiated by the TGF-β signaling pathway, and further activated MMP and cathepsin secretion to induce the degradation of ECM components. This study may provide a therapeutic strategy to decrease collagen accumulation in the skeletal muscle of elderly individuals and recover muscle functions.

## 2. Results

### 2.1. Alteration of ECM Components in Skeletal Muscle with Age

**Figure 1:**
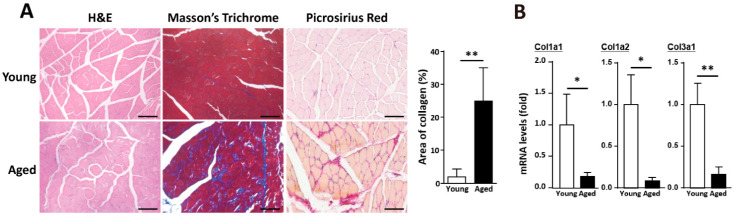
Collagen accumulation in aged skeletal muscle is accompanied by the downregulation of collagen genes. (**A**) Histological results of young and aged muscle, including hematoxylin-eosin (H&E) staining, Masson’s trichrome staining, and picrosirius red staining. Quantitative data of collagen area (blue part) from Masson’s Trichrome staining in young and aged skeletal muscle. At least 5 captured images of muscle sections from different fields in each mouse sample were examined, and there were 3 mice in each group. Scale bar, 100 µm. (**B**) Quantitative RT-qPCR messenger RNA (mRNA) expression data of collagen genes in young and aged skeletal muscle. Five mice were used in each group. The results are presented as the mean ± SD. * *p* < 0.05; ** *p* < 0.005.

In aged skeletal muscle, a decline in regeneration leads to the accumulation of adipose tissues and collagen. Mature type I collagen (in the perimysium) is produced from the Col1a1 and Col1a2 genes, while mature type III collagen (in the endomysium and the epimysium) is produced by the Col3a1 gene [23]. Collagen accumulation was observed in muscle fibers, including the endomysium and perimysium, in the aged skeletal muscles compared with the young skeletal muscles (Figure 1A). However, the gene expression levels of fibrillar collagen (collagens I and III) were significantly lower in the aged skeletal muscle than the young skeletal muscle (Figure 1B). Therefore, collagen deposition in the ECM of aged muscle was not a direct consequence of elevated fibrillar collagen expression. To investigate biological changes in the ECM of aged muscle, we determined the expression levels of different muscle ECM components by transcriptomic analysis. Based on our previous report [24], in the aged skeletal muscle, fibrillar collagens (collagens I and III) were dramatically downregulated (Table 1), and other critical components of the ECM, such as networking collagens (collagens IV and VI), laminin, fibronectin, elastin, nidogen, heparan, and biglycan, were also downregulated in the aged muscle (Table 1). The gene expression levels of collagen and other ECM components were downregulated in the aged skeletal muscle, which indicated decreased production of these proteins.

In addition to the structural/biological changes, the expression of secreted factors in the skeletal muscles was examined during the aging process. Matricellular proteins that were secreted to muscle ECM to maintain the architecture, such as tenascin-C, secreted acidic cysteine rich glycoprotein (SPARC), WNT1-inducible-signaling pathway protein 1 (WISP1) and thrombospondin, were downregulated in the aged skeletal muscle (Table 1). These matricellular proteins are important for ECM signaling and organization, although they do not directly provide structural support in the ECM. Therefore, we hypothesized that the accumulation was due to poor clearance of excessive ECM components with age.

### 2.2. An Age-Dependent Decline in the Enzymes Involved in ECM Remodeling

**Figure 2:**
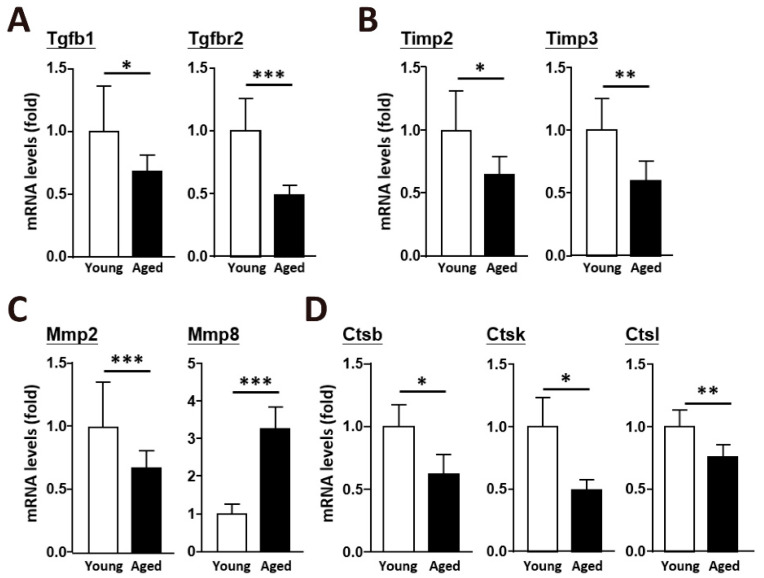
During natural aging, TGF-β signaling pathways were downregulated in skeletal muscle. Quantitative data of RT-qPCR mRNA expression of (**A**) TGF-β genes, (**B**) Timp genes, (**C**) Mmp genes, and (**D**) cathepsin genes in young and aged skeletal muscle. There were 8 mice in each group, and all of the mice were not subjected to any form of muscle injury. The results are presented as the mean ± SD. * *p* < 0.05; ** *p* < 0.005; *** *p* < 0.001.

To elucidate the mechanism of fibrillar collagen regulation in the ECM of aged muscle, we investigated the underlying molecular pathway. Several signaling transduction pathways are involved, and the TGF-β pathway is crucial for regulating the expression of type I and type III collagens. Transforming growth factor beta 1 (TGFb1) and transforming growth factor beta receptor II (TGFbr2) were downregulated in the aged skeletal muscle (Figure 2A). ECM integrity and remodeling depend on the dynamic balance between MMPs and TIMPs. The expression of Timp2 and Timp3, which activate pro-mmps to generate Mmps, was significantly decreased in aged mice (Figure 2B), and the predominant metalloproteinase in skeletal muscle, Mmp2, was decreased as well (Figure 2C). Proteolytic cleavage by cysteine cathepsins represents another mechanism involved in ECM remodeling. Cathepsins B, K, and L, which are expressed in skeletal muscle (Figure 2D), were downregulated in the aged skeletal muscle. According to a transcriptomic study, TGFb1, TGFbr2 and their related growth factors are downregulated in aged skeletal muscle. Mmp2, Mmp3, Timp1, Timp2, and Timp3 were also decreased, indicating a reduced ability to cleave ECM components with age (Table 1). In addition, a transcriptomic study showed decreased expression of Stat3 and Stat6 (Table 1), which activate the signal transducers and activators of transcription (STAT) transcription pathways to induce cathepsin secretion in aged skeletal muscle.

### 2.3. Clearance of Accumulated Collagen in Aged Skeletal Muscle after Muscle Injury

**Figure 3:**
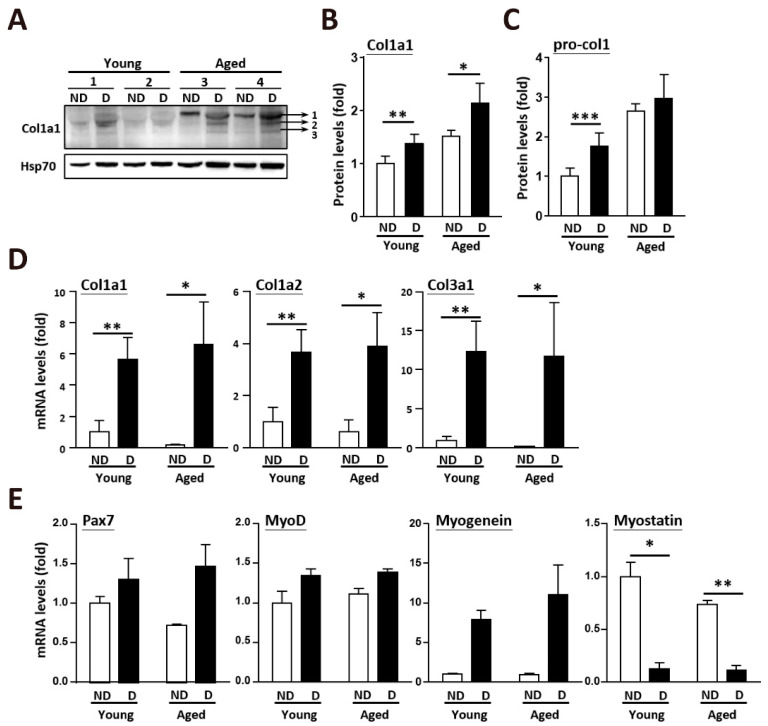
After muscle injury, the expression levels of collagen genes were upregulated, and collagen deposition in aged muscle was diminished. (**A**) Protein level of col1a1 expression in young and old muscle after damage. ND, nondamaged muscle which was from contralateral leg of the damaged muscle as a control. D, fourteen days after BaCl_2_ muscle injury. (**B**) Quantitative results of collagen 1a1 (bands 1, 2, and 3 in total from Figure 3A) expression in young and aged muscle. There were 3 mice in each group. (**C**) Quantitative results of pro-collagen (band 1 in Figure 3A) expression in young and aged muscle. There were 3 mice in each group. (**D**) Quantitative RT-qPCR mRNA expression of collagen genes in young and aged skeletal muscle after damage. Five mice in each group. (**E**) Quantitative data of RT-qPCR mRNA expression of myogenesis-related genes in young and aged skeletal muscle after damage. The results are presented as the mean ± SD. * *p* < 0.05; ** *p* < 0.005; *** *p* < 0.001.

**Figure 4:**
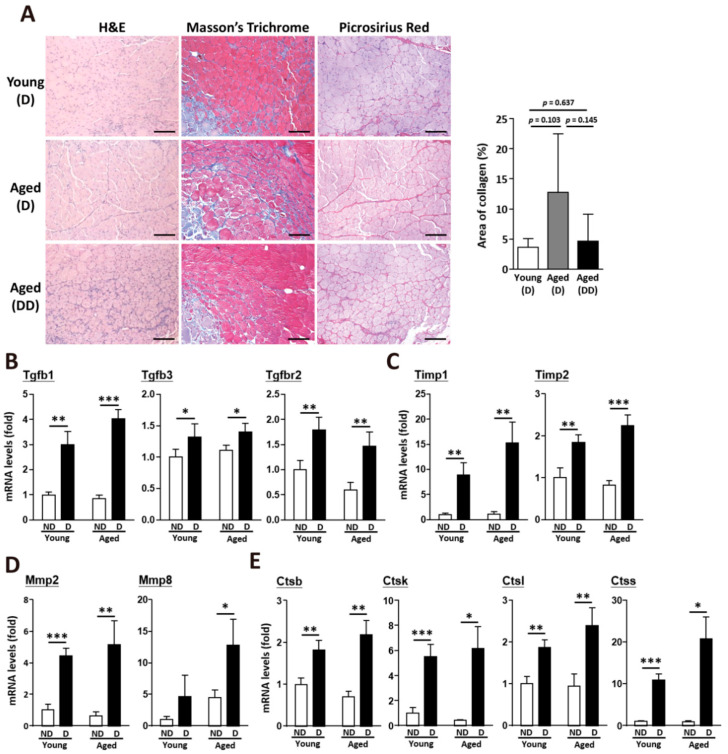
Upregulation of TGF-β signaling activated ECM remodeling after muscle injury. (**A**) Histological results of young and aged skeletal muscle after muscle injury, including H&E staining, Masson’s trichrome staining, and picrosirius red staining. Muscle in the aged double dose (dd) group was damaged twice at day 0 and day 7. Quantitative data of collagen area (blue part) from Masson’s Trichrome staining results in young and aged skeletal muscle. At least 5 captured images of muscle sections from different fields in each mouse sample were examined, and there were 3 mice from each group. Scale bar, 100 µm. (**B**) Quantitative data of RT-qPCR mRNA expression of TGF-β, (**C**) Timp genes, (**D**) Mmp genes, and (**E**) cathepsin genes in young and aged skeletal muscle. There were 4 mice in each group. ND, nondamaged muscle as a control. D, Fourteen days after BaCl_2_ muscle injury. The results are presented as the mean ± SD. * *p* < 0.05; ** *p* < 0.005; *** *p* < 0.001.

After muscle injury, the collagen levels were dramatically increased to fill the damaged area. Total collagen type I alpha 1 (Col1α1) was substantially increased in the aged skeletal muscle after injury (Figure 3A). In the undamaged skeletal muscle, Col1a1 was approximately 1.5-fold higher in the aged muscle than in the young muscle. After muscle injury, both the young and aged muscles showed increases in total Col1a1 and procollagen (Figure 3B,C). Additionally, the messenger RNA (mRNA) levels of fibrillar collagens (collagen I and III) were strongly upregulated (Figure 3D). The alteration of myogenesis-related genes was caused by muscle injury, but not age issues (Figure 3E). However, two weeks after injury, when regenerated muscle fibers (fibers with centrally located nuclei) were generated, collagen accumulation in the muscle was eliminated, even in the aged skeletal muscle (Figure 4A). After muscle damage, collagen accumulation increased about 1.78-fold in the young mice; however, collagen accumulation decreased about 1.94-fold in the aged mice (young after damage, 3.61% ± 1.45%; aged after damage, 12.77% ± 9.75%). More interestingly, collagen accumulation dramatically decreased about 5.37-fold in the aged mice after double muscle injuries (aged after double damage, 4.66% ± 4.42%). Following acute muscle injury, the expression of genes that regulate the balance of ECM components, including Tgfb1, Tgfb3, and Tgfbr2, was upregulated in the young and aged muscles (Figure 4B). TIMPs activated by the TGF-β pathway, such as Timp1 and Timp2 (Figure 4C), were also upregulated after muscle regeneration, which further activated Mmp2 and Mmp8 (Figure 4D) for the degradation and remodeling of the ECM. The elevated MMP activities, which occurred as part of the inflammatory phase of healing, were likely beneficial in the repair and regeneration of damaged ECM. However, the expression of the cysteine proteases cathepsin B, K, L, and S was also increased at 14 days post-injury (Figure 4E).

### 2.4. Possible Mechanisms of the Effects of Aging on ECM Components in Skeletal Muscle

**Figure 5:**
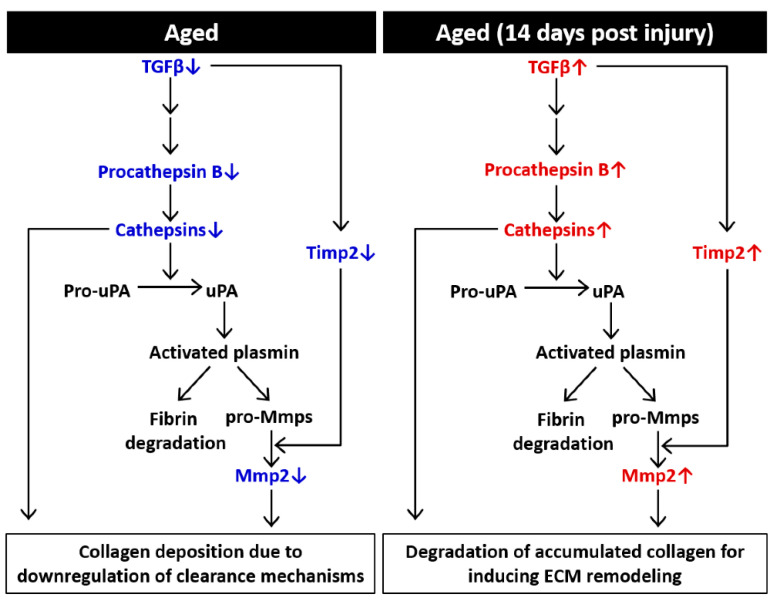
Schematic diagram showing ECM-mediated effects on remodeling in aged muscle. In aged skeletal muscle, downregulated TGF-β signaling triggers transcriptional changes, including downregulation of procathepsin B and Timp2, which participate in further ECM remodeling. Cathepsins also activate pro-uPA, initiating a uPA-mediated cascade of proteolytic cleavage that results in the activation of plasmin, fibrin and pro-MMPs. After muscle injury, TGF-β induces subsequent transcriptional changes that lead to ECM remodeling, further degradation of collagen and transient deposition of ECM. uPA, urokinase plasminogen activator.

In conclusion, in aged skeletal muscle, we suggested that a decrease in TGF-β expression downregulated TIMPs and cathepsins, which further inhibited the effective degradation of ECM components (Figure 5). This mechanism may explain why collagen accumulation is higher in the aged skeletal muscle than young skeletal muscle. After acute muscle injury, both young and aged muscles underwent complete repair and regeneration. Although the expression levels of fibrillar collagen genes were increased, the upregulated TGF-β-induced signaling pathway could activate downstream ECM remodeling enzymes, including MMPs and cathepsins, to eliminate the accumulation of ECM components. These data support the hypothesis that age-associated changes in ECM components might be driven by decreased degradation rather than by the increased synthesis of collagenous structures.

## 3. Discussion

Aged skeletal muscle shows thickening of the ECM and typically demonstrates fibrotic morphology [25,26]. Sarcopenia is characterized by the loss of muscle mass and force accompanied by increased fibrosis [27]. Age-associated fibrosis is regulated by different factors, such as defects in cell populations and changes in cell signaling and growth factors, which in turn lead to changes in the muscle microenvironment [28]. In previous reports, age-associated muscle fibrosis is clinically relevant, especially for the mechanical impact of ECM on muscle function, and is characterized by the loss of healthy perimysial collagen fibers and an increase in ECM proteins (collagen), as well as the accumulation of debris in the endomysium and perimysium due to impaired protein degradation [29,30].

Following acute muscle injury, MuSCs are activated to promote repair and regeneration; in addition, the accumulation of endomysial connective tissue in both humans and rodents has been confirmed, and is usually resolved as muscle regenerates [30,31]. In the case of severe injury, the effective resolution of fibrosis accompanied by collagen remodeling is required for the restoration of the muscle to the preinjury state. Proteolytic cleavage and the clearance of ECM components and matricellular proteins occur when the ECM is remodeled after injury. Several families of proteases function in the ECM, including MMPs, cysteine proteases and serine proteases. Proteases such as those in the MMP, serine protease and cysteine protease families influence the integrity and dynamic balance of the ECM at multiple levels [32,33]. MMPs are the main proteinases that break down collagen in the ECM and are inhibited by TIMPs [34,35]. In skeletal muscle, the gelatinase MMP-2 and membrane-bound MMP-14 constitute more than 90% of the total MMPs and may be key regulators of ECM degradation in skeletal muscle [36]. There are four kinds of TIMPs, TIMP-1 through TIMP-4, and all of them are expressed in skeletal muscle and can inhibit all known MMPs [23,37]. Extracellular cathepsins have been shown to participate in ECM remodeling by degrading abundant structural ECM components (e.g., collagen or elastin) [38]. ECM proteolysis is also regulated by the interaction between cathepsins and the urokinase-type plasminogen activator and its receptor (uPA-uPAR). Active uPA activates plasmin, which cleaves ECM proteins and activates pro-MMPs [39]. Other studies have also demonstrated that pro-uPA and uPAR, in complex with uPAR-associated protein (uPARAP), mediate the endocytosis of extracellular collagen [40].

The mechanisms of age-induced changes in muscle ECM are still unclear. These phenomena could be driven by the excessive synthesis of ECM collagenous components, altered expression and activities of ECM-degrading enzymes and their inhibitors, or a combination of these factors [41]. In our study, aging typically led to the increased deposition of collagenous tissue in skeletal muscle, and these changes were basically due to decreased MMP and cathepsin activities. In muscle injury, which impairs the muscle structure, the aged muscle displayed lower regenerative potential than the young muscle; however, this impairment induced MMP and cathepsin activities, and collagen deposition in aged skeletal muscle was partially recovered. These data are consistent with previous work showing that lower levels of elastin and fibronectin in aged muscle lead to compromised muscle maintenance and the degradation of the ECM through tissue necrosis [42]. During the aging process, one of the major cellular proteolytic mechanisms mediated by the lysosome is impaired. The autophagy pathways with lysosomes and cathepsins play a critical role in the degradation in the last step of autophagy. Therefore, enhancing the activity of cathepsins may help to clean the misfolded and accumulated ECM and promote ECM remodeling [43].

A group of matricellular proteins, including SPARC, thrombospondin, and tenascin-C, are secreted into the ECM of skeletal muscle. SPARC has been shown to bind collagen and plays a role in maintaining the stiffness of skeletal muscle by regulating collagen accumulation as a myokine [44,45]. Thrombospondin has been identified in the ECM of skeletal muscle, and collagen fibrils were shown to be disorganized in multiple tissues of thrombospondin knockout mice [46]. Tenascin-C is localized to the neuromuscular junction (NMJ) and binds perlecan and agrin [47], which participates in maintaining NMJ architecture [48]. The temporary upregulation of tenascin-C and fibronectin results in transient matrix, which is considered an essential first step for successful muscle repair [49,50]. Aging also disrupts MuSC function by impairing the secretion of the matricellular protein WISP1 from fibro/adipogenic progenitors [51]. Previous research showed that following acute exercise and training, skeletal muscle had many regulated ECM proteins, including several collagens, proteoglycans, and modulators of the ECM, such as cathepsins and MMPs [52,53,54]. Moreover, after acute exercise, the mRNA abundance of TGFb1 and TGFbr2 was increased, indicating that TGF-β contributes to the adaptation of the ECM to exercise [55]. With respect to serine protease and cathepsins, the upregulation of cathepsin B expression has been reported in myeloid tumor cell lines following TGF-β1 treatment, which identified a link between TGF-β1 signaling and the degradation of ECM components [56].

For the inhibition of muscle fibrosis or excessive collagen accumulation, targeting the TGF-β signaling pathway to induce ECM clearance may be an important strategy for clinical use in the future. Several TGF-β inhibitors have been discussed and reviewed [57]. Several reports studied on aged human individuals indicated that exercise could help to alter the expression of ECM genes and ECM remodeling enzymes and may reduce age-associated muscle fibrosis [32,58,59]. In this respect, exercise training adaptation or other methods to induce mild skeletal muscle injury, which could further enhance the activities of myokines or ECM remodeling enzymes, can be used as potential therapeutic strategies to restore muscle functions in elderly individuals.

The current study still has some interpretation limitations. All mice in this study were male; therefore, we could not know whether female mice have a similar situation on skeletal muscles under aged and/or damaged conditions. In order to investigate the regenerated capacity of muscle stem cells, we used a BaCl_2_-injuried model. BaCl_2_ injures the myofibers, but not satellite cells, due to blocking potassium channels and overloading intracellular calcium, resulting in the apoptosis of myofibers. However, we could not validate whether the results in this study can apply to every injury model. Although we demonstrated that initiating regeneration could drive ECM remodeling for decreasing collagens which accumulate in skeletal muscles, the detailed mechanisms are needed to evaluate in the future.

## 4. Materials and Methods

### 4.1. Mice

Wild-type male mice (C57BL/6) at 3–5 (young group) and 20–24 (old group) months of age were maintained in a pathogen-free facility. There were at least three mice in each group for following analysis. To induce muscle injury, fifty microliters of 1.2% BaCl_2_ (342920, Sigma Aldrich, St. Louis, MO, USA) in normal saline was injected into the right leg gastrocnemius muscle after anesthesia by intraperitoneal injection of 2.5% Avertin (Sigma, T48402) at 15 µL per gram of body weight. The contralateral leg served as a control. After muscle injury, the damaged muscle was harvested on day 14. Muscle in the aged double dose (dd) group was damaged twice at day 0 and day 7. The animal protocol was approved by the Institutional Animal Care and Use Committee of Taipei Medical University and National Defense Medical Center.

### 4.2. RNA Analysis

RNA was extracted from muscle tissue using TRIzol™ Reagent (15596018, Invitrogen™, Carlsbad, CA, USA) according to the manufacturer’s instructions, and complementary DNA (cDNA) was synthesized from 2 µg total RNA of muscle tissues using a High-Capacity cDNA Reverse Transcription Kit (4368813, Applied Biosystems™, Waltham, MA, USA). RT-qPCR was performed with specific primers (Supplemental Table S1) and TaqMan™ Universal Master Mix II, no UNG (Applied Biosystems™, 4440040), or Power SYBR™ Green PCR Master Mix (Applied Biosystems™, 4367359). PCR reactions were run on a QuantStudio3 Real-Time PCR system (Applied Biosystems) under standard conditions. All amplifications were carried out in triplicate for each RNA sample and primer set. The amount of total input cDNA was calculated using hypoxanthine guanine phosphoribosyl transferase (Hprt) as an internal control for both TaqMan™ and SYBR™ Green master mix.

### 4.3. Protein Analysis

Muscle samples were homogenized in Radioimmunoprecipitation assay buffer (RIPA buffer) (50 mM Tris at pH 7.4, 150 mM NaCl, 1% Triton X-100, 1% SDS, 1% deoxycholate with complete protease inhibitor cocktail (04693132001, Roche, Penzberg, Germany). The extracted protein was separated on a 4–12% NuPAGE™ Bis-Tris protein gel (Invitrogen™, NP0323BOX) without boiling in NuPAGE™ MOPS SDS Running Buffer (Invitrogen™, NP0001) and electrotransferred to an Amersham™ Hybond^®^ *p* Western blotting membrane (10600023, GE Healthcare, Chicago, IL, USA). The membranes were blocked with 5% (*w*/*v*) nonfat dry milk and 5% (*w*/*v*) bovine serum albumin (Sigma, A7906) and incubated with primary antibodies against Col1a1 (1:1000, ab34710, Abcam, Berlin, Germany) and Hsp70 (1:5000, GTX111088, GeneTex, Irvine, CA). The data were detected using a Visualizer Kit (WBKLS0500, Millipore, Burlington, MA, USA). Hsp70 was used as an internal control for each sample in protein analysis, and quantitative protein levels were expressed by relative fold changes.

### 4.4. Histological Analysis

Skeletal muscle was collected and fixed with 10% neutral buffered formalin (Burnett) and subsequently embedded in paraffin. Three-micrometer paraffin sections were subjected to hematoxylin-eosin (H&E) staining, Masson’s trichrome staining (Muto Pure Chemical Co., Tokyo, Japan), and picrosirius red staining (24901, Polysciences Inc., Warrington, PA, USA) by standard procedures. Slides were visualized with an IX73 microscope (Olympus) equipped with a Q Imaging Retina 3000 camera and Q Capture Pro 7 software to capture images. In Masson’s trichrome images, according to the manufacturer’s instructions, we quantified the blue part, which indicates the staining of collagen, of each image using ImageJ software. Taking separation due to the use of paraffin-embedded sections into consideration, we calculated the percentage of collagen area to total muscles excluding the separation part in each image at the same time.

### 4.5. Statistical Aanalysis

The results are presented as the mean ± SD of at least three independent samples. Comparisons between two groups were carried out using two-tailed, unpaired Welch *t*-tests, and the *p*-values were calculated. Statistical differences were considered significant when *p* < 0.05. Asterisks indicate *p < 0.05; **p < 0.01; and ***p < 0.001.

## Figures and Tables

**Table 1:** **Transcriptomic study results.** Expression of selected genes associated with ECM components, ECM remodeling, growth factors in young and aged skeletal muscle.

chr	Gene	Name	SM-Young	SM-Aged	Aged to Young (logfc)
**Gene Expression Involved in Fibrillar and Fibril-Associated Collagen**					
chr6	*Col1a2*	ENSMUSG00000029661.9	506.20	188.90	−1.418
chr11	*Col1a1*	ENSMUSG00000001506.9	252.60	88.43	−1.504
chr15	*Col2a1*	ENSMUSG00000022483.9	0.24	0.20	−0.045
chr1	*Col3a1*	ENSMUSG00000026043.11	604.40	224.10	−1.427
chr2	*Col5a1*	ENSMUSG00000026837.9	65.31	44.19	−0.553
chr1	*Col5a2*	ENSMUSG00000026042.9	69.95	44.04	−0.656
chr3	*Col11a1*	ENSMUSG00000027966.10	5.08	1.25	−1.435
chr4	*Col27a1*	ENSMUSG00000045672.7	6.59	5.29	−0.272
chr9	*Col12a1*	ENSMUSG00000032332.10	18.83	8.73	−1.027
chr15	*Col14a1*	ENSMUSG00000022371.8	25.70	19.70	−0.367
**Gene Expression Involved in Networking Collagen**					
chr8	*Col4a1*	ENSMUSG00000031502.9	202.00	191.70	−0.075
chr1	*Col4a4*	ENSMUSG00000067158.7	5.22	4.74	−0.116
chrX	*Col4a5*	ENSMUSG00000031274.9	13.42	6.98	−0.853
chr10	*Col6a1*	ENSMUSG00000001119.6	116.50	85.94	−0.434
chr10	*Col6a2*	ENSMUSG00000020241.7	105.40	79.30	−0.406
chr1	*Col6a3*	ENSMUSG00000048126.9	113.20	76.96	−0.550
chr9	*Col6a4*	ENSMUSG00000032572.5	1.80	0.00	−1.487
chr9	*Col6a5*	ENSMUSG00000091345.1	2.23	1.20	−0.556
chr10	*Col18a1*	ENSMUSG00000001435.8	26.61	20.90	−0.334
**Down-Regulation of the Gene Expression which Encodes BM Glycoproteins**					
chr2	*Lamc3*	ENSMUSG00000026840.8	4.17	4.14	−0.009
chr12	*Lamb1*	ENSMUSG00000002900.8	57.48	57.06	−0.010
chr17	*Lama1*	ENSMUSG00000032796.7	0.09	0.05	−0.061
chr10	*Lama2*	ENSMUSG00000019899.8	80.81	67.73	−0.251
chr1	*Lamc1*	ENSMUSG00000026478.8	180.50	150.80	−0.258
chr1	*Fn1*	ENSMUSG00000026193.8	210.20	122.10	−0.779
chr5	*Eln*	ENSMUSG00000029675.9	27.27	23.69	−0.195
chr14	*Nid2*	ENSMUSG00000021806.3	18.83	18.55	−0.020
chr13	*Nid1*	ENSMUSG00000005397.7	217.50	161.70	−0.425
**Down-Regulation of the Gene Expression which Encodes Proteoglycans and GAGs**					
chr4	*Hspg2*	ENSMUSG00000028763.10	267.70	263.70	−0.022
chr10	*Dcn*	ENSMUSG00000019929.8	892.00	792.50	−0.171
chrX	*Bgn*	ENSMUSG00000031375.10	113.70	76.01	−0.575
chr1	*Fmod*	ENSMUSG00000041559.7	152.20	82.30	−0.879
**Down-Regulation of the Gene Expression which Encodes Growth Factors**					
chr7	*Tgfb1*	ENSMUSG00000002603.9	12.62	7.73	−0.641
chr9	*Tgfbr2*	ENSMUSG00000032440.5	78.77	68.03	−0.209
chr10	*Igf1*	ENSMUSG00000020053.11	64.03	36.46	−0.796
chr10	*Ctgf*	ENSMUSG00000019997.4	40.17	29.33	−0.441
**Gene Expression which Encodes Matrix Matricellular Proteins**					
chr11	*Sparc*	ENSMUSG00000018593.6	583.80	351.50	−0.730
chr2	*Thbs1*	ENSMUSG00000040152.8	15.98	23.54	0.531
chr17	*Thbs2*	ENSMUSG00000023885.7	35.19	34.12	−0.043
chr3	*Thbs3*	ENSMUSG00000028047.5	10.72	8.98	−0.232
chr13	*Thbs4*	ENSMUSG00000021702.7	160.50	100.70	−0.667
chr4	*Tnc*	ENSMUSG00000028364.8	11.10	4.79	−1.064
chr5	*Spp1*	ENSMUSG00000029304.8	4.51	10.67	1.084
chr15	*Wisp1*	ENSMUSG00000005124.3	1.47	0.50	−0.721
**Gene Expression which Encodes Cathepsins**					
chr3	*Ctsk*	ENSMUSG00000028111.3	14.28	9.28	−0.572
chr13	*Ctsl*	ENSMUSG00000021477.7	153.80	149.50	−0.040
chr14	*Ctsb*	ENSMUSG00000021939.6	523.70	435.90	−0.264
chr3	*Ctss*	ENSMUSG00000038642.4	40.98	15.56	−1.342
chr2	*Ctsz*	ENSMUSG00000016256.9	52.07	42.69	−0.281
chr7	*Ctsc*	ENSMUSG00000030560.9	95.28	74.07	−0.359
chr19	*Ctsf*	ENSMUSG00000083282.2	31.87	28.53	−0.155
chr9	*Ctsh*	ENSMUSG00000032359.8	34.95	18.95	−0.850
chr3	*Ctso*	ENSMUSG00000028015.2	20.91	19.90	−0.068
chr19	*Ctsw*	ENSMUSG00000024910.4	2.09	0.10	−1.489
chr11	*Stat3*	ENSMUSG00000004040.10	95.90	93.57	−0.035
chr10	*Stat6*	ENSMUSG00000002147.11	47.33	41.15	−0.198
**Gene Expression which Encodes Matrix Remodeling Enzymes**					
chr9	*Mmp3*	ENSMUSG00000043613.7	9.39	3.24	−1.292
chr8	*Mmp2*	ENSMUSG00000031740.7	95.14	61.30	−0.626
chrX	*Timp1*	ENSMUSG00000001131.5	0.95	0.75	−0.157
chr11	*Timp2*	ENSMUSG00000017466.3	261.40	226.70	−0.205
chr10	*Timp3*	ENSMUSG00000020044.7	184.30	177.20	−0.057

SM, skeletal muscle.

## Data Availability

The dataset analyzed in the current study is openly available from the ArrayExpress repository (E-MTAB-5176), https://www.ebi.ac.uk/arrayexpress/experiments/E-MTAB-5176 (accessed on 15 January 2021).

## Supplementary Materials

The following are available online at https://www.mdpi.com/1422-0067/22/4/2123/s1, Table S1: Primer list.
