# Supplementary material for: Aged Skeletal Muscle Retains the Ability to Remodel Extracellular Matrix for Degradation of Collagen Deposition after Muscle Injury
Source: Int J Mol Sci. 2021 Feb 20;22(4):2123. doi: 10.3390/ijms22042123 (PMC7924602; doi:10.3390/ijms22042123)
Supplement: Supplementary file 1 [file ijms-22-02123-s001.pdf]

Supplemental Table S1

Supplemental Table S1. Primer list. Primers were designed and performed on quantification of specific mouse gene expression.

Primers for qPCR with TaqMan™ system

Official Symbol	Name	Sequence
Hprt	hypoxanthine guanine phosphoribosyl transferase	F: 5'-CCTCCTCAGACCGCTTTTT-3'
		R: 5'-AACCTGGTTCATCATCGCTAA-3'
Col1a1	Collagen, type I, alpha 1	F: 5'-CATGTTCAGCTTTGTGGACCT-3'
		R: 5'-GCAGCTGACTTCAGGGATGT-3'
Col1a2	Collagen, type I, alpha 2	F: 5'-CAAGCATGTCTGGTTAGGAGAG-3'
		R: 5'-AGGACACCCCTTCTACGTTGT-3'
Col3a1	Collagen, type III, alpha 1	F: 5'-TTCCAGGACAACCAGGTCTC-3'
		R: 5'-AGTCGAATTGGGGAGAATAATTT-3'
Mmp2	Matrix metallopeptidase 2	F: 5'-GTGGGACAAGAACCAGATCAC-3'
		R: 5'-GCATCATCCACGGTTTCAG-3'
Mmp8	Matrix metallopeptidase 8	F: 5'-AACGGGAAGACATACTTCTTCATAA-3'
		R: 5'-GGGTCCATGGATCTTCTTTG-3'
Mmp9	Matrix metallopeptidase 9	F: 5'-AGACGACATAGACGGCATCC-3'
		R: 5'-TCGGCTGTGGTTCAGTTGT-3'
Timp1	Tissue inhibitor of metalloproteinase 1	F: 5'-GCAAAGAGCTTTCTCAAAGAC-3'
		R: 5'-AGGGATAGATAAACAGGGAAACACT-3'
Timp2	Tissue inhibitor of metalloproteinase 2	F: 5'-CGTTTTGCAATGCAGACGTA-3'
		R: 5'-GGAATCCACCTCCTTCTCG-3'
Timp3	Tissue inhibitor of metalloproteinase 3	F: 5'-CCACGTGCAGTACATTCACAC-3'
		R: 5'-CCTGTCAGCAGGTACTGGTATTT-3'
Timp4	Tissue inhibitor of metalloproteinase 4	F: 5'-CTGAGGCTGCTGGCTTTG-3'
		R: 5'-GGATATTTTGGCCCGTATCA-3'
Ctsb	Cathepsin B	F: 5'-CCGACCATTGGACAGATTAGA-3'
		R: 5'-CTTCCACTGCCCCAAATG-3'
Ctsk	Cathepsin K	F: 5'-CTCCATCGACTATCGAAAGAAAG-3'
		R: 5'-AAAGCCCAACAGGAACCAC-3'
Ctsl	Cathepsin L	F: 5'-AAGGGTTGTGTGACTCCTGTG-3'
		R: 5'-CAACCCGATGCGCTAAAC-3'

Ctss	Cathepsin S	F: 5’-CATCTTTGGAGTGAGCACCA-3’
		R: 5’-GCATCCAAAACAGCCATCTTA-3’

Primers for qPCR with SYBR® Green system

Official Symbol	Name	Sequence
Hprt	hypoxanthine guanine phosphoribosyl transferase	F: 5’-TGAAAGACTTGCTCGAGATGTCA-3’
		R: 5’-CACACAGAGGGCCACAATGT-3’
Tgfb1	Transforming growth factor, beta 1	F: 5’-GCAGTGGCTGAACCAAGGA-3’
		R: 5’-AGCAGTGAGCGCTGAATCG-3’
Tgfb2	Transforming growth factor, beta 2	F: 5’-AGAGCTCGAGGCGAGATTTG-3’
		R: 5’-TTCTGATCACCACTGGCATATGT-3’
Tgfb3	transforming growth factor, beta 3	F: 5’-GTGTACGCCCCCTTTATATTGACT-3’
		R: 5’-GGTTCGTGGACCCATTTCC-3’
Tgfbr1	transforming growth factor, beta receptor I	F: 5’-GTGCCTTGAGGCCCTGTGT-3’
		R: 5’-CTGCAGCGAGAACCAAAATCT-3’
Tgfbr2	transforming growth factor, beta receptor II	F: 5’-ACGAGCCCCCATTTGGTT-3’
		R: 5’-CTCAGCACACTGTCTTTCATGCT-3’
